# PPAR**γ** Agonists in Adaptive Immunity: What Do Immune Disorders and Their Models Have to Tell Us?

**DOI:** 10.1155/2013/519724

**Published:** 2013-08-01

**Authors:** Laurindo Ferreira da Rocha Junior, Andréa Tavares Dantas, Ângela Luzia Branco Pinto Duarte, Moacyr Jesus Barreto de Melo Rego, Ivan da Rocha Pitta, Maira Galdino da Rocha Pitta

**Affiliations:** ^1^Serviço de Reumatologia do Hospital das Clínicas da Universidade Federal de Pernambuco (HC-UFPE), Recife 50670-901, PE, Brazil; ^2^Laboratório de Imunomodulação e Novas Abordagens Terapêuticas (LINAT), Núcleo de Pesquisa em Inovação Terapêutica (NUPIT SG), Universidade Federal de Pernambuco (UFPE), Recife 50670-901, PE, Brazil

## Abstract

Adaptive immunity has evolved as a very powerful and highly specialized tool of host defense. Its classical protagonists are lymphocytes of the T- and B-cell lineage. Cytokines and chemokines play a key role as effector mechanisms of the adaptive immunity. Some autoimmune and inflammatory diseases are caused by disturbance of the adaptive immune system. Recent advances in understanding the pathogenesis of autoimmune diseases have led to research on new molecular and therapeutic targets. PPAR**γ** are members of the nuclear receptor superfamily and are transcription factors involved in lipid metabolism as well as innate and adaptive immunity. PPAR**γ** is activated by synthetic and endogenous ligands. Previous studies have shown that PPAR agonists regulate T-cell survival, activation and T helper cell differentiation into effector subsets: Th1, Th2, Th17, and Tregs. PPAR**γ** has also been associated with B cells. The present review addresses these issues by placing PPAR**γ** agonists in the context of adaptive immune responses and the relation of the activation of these receptors with the expression of cytokines involved in adaptive immunity.

## 1. Introduction

Adaptive immunity is a very powerful and specialized tool of host defense. The T- and B-cell lymphocytes are classically involved in the adaptive immune system. Disturbances of the adaptive immunity results in autoimmunity. Immune dysfunction associated with autoimmune diseases was known to be caused by an imbalance between Th1 and Th2 cells. Autoimmune diseases could be categorized as predominantly Th1-driven if the major events were cell mediated in nature, or predominantly Th2 driven if antibodies and/or immune complexes served as the main mediators [1]. In the last years, a third subset named Th17 cells has been identified, and the Th1/Th2 imbalance hypothesis has shifted to an involvement of the Th1/Th2/Th17/regulatory T (Treg) lymphocytes with the same Th precursor cells [2]. B-cell activation and antibody production can be either an independent T-cell help process or B cells receiving help from follicular T cells. In autoimmune diseases, the contact with self-antigen leads to B-cell activation and, therefore, these lineage of cells are of great importance in adaptive immunity. Naive B cells develop into antibody-producing plasma cells through the contact with antigen in combination with TLR-agonists and cytokines. Activation of B cells also results in differentiation into plasma blasts and increased cytokine production [3].

The nuclear receptor (NR) superfamily is composed of 48 members and includes receptors for steroid hormones, thyroid hormone, various lipids, and oxysterols. NRs function as ligand-dependent transcription factors and share a modular domain structure [4]. PPAR-gamma (PPAR*γ*) belongs to the nuclear receptor superfamily. These transcription factors function as receptors for various lipid-soluble, small molecules that are most commonly generated as hormones or in the intermediary metabolic pathways [5]. These receptors regulate gene expression upon heterodimerization with the retinoid X receptor by ligating to peroxisome proliferator response elements (PPREs) in the promoter region of target genes. These genes are regulated through ligand-dependent transcriptional activation. Several of these target genes are involved in metabolic homeostasis [6]. The PPAR*γ* express two isoforms, PPAR-*γ*1 and PPAR-*γ*2. PPAR-*γ*1 is expressed in macrophages, colonic epithelial cells, endothelial cells, and vascular smooth muscle cells. PPAR-*γ*2 is mainly expressed in adipose tissue and is involved in the regulation of adipogenesis [7]. PPAR*γ* activation in immune cells predominantly results in another mechanism of action: transrepression of proinflammatory gene expression [8]. Transrepression does not involve binding of the nuclear receptor to its cognate DNA element, but here PPAR*γ* operates by antagonizing signal-dependent activation of its target genes by other classes of transcription factors, including NF-*κ*B and AP-1 proteins, thereby reducing inflammatory signaling pathways [9]. PPAR*γ* expression in the monocyte/macrophage lineage was demonstrated by the suppression of the activation of monocyte/macrophages by PPAR*γ* agonists. In Addition to their role in the anti-inflammatory response of innate immune cells, PPARs are involved in mediating the adaptive immune responses of T and B cells [10]. PPAR*γ* is activated by diverse synthetic and naturally occurring substances. Many ligands that activate and modulate PPAR functions have been identified [10]. Naïve and activated T cells express PPAR*γ*, and ligands for the receptor inhibit proliferation and significantly decrease cell viability [11]. Activated B cells upregulate their expression of PPAR*γ* [12]. In this review, we will summarize the recent progress in PPAR*γ* studies and the interplay of these nuclear receptors with adaptive immunity and T and B cells.

## 2. Th1 Lymphocytes

Th1 cells secrete interferon- (IFN-) *γ*, interleukin- (IL-) 2, and tumor necrosis factor (TNF) and control protection against infection with intracellular microbes. Maturation of Th1 cells is controlled by IL-12 and transcription factor T-bet. During Th1 cell differentiation, IL-12 signals via the IL-12R/STAT4-signalling pathway inducing IFN-*γ* expression, the secreted IFN-*γ* then signals through the IFN-*γ*R/STAT1 pathway to further increase IFN-*γ* levels, forming a positive autoregulatory loop reinforcing Th1 differentiation. The signal transducer and activator (STAT) proteins STAT1 and STAT4 induce the expression of the Th1-specific transcription factor T-box expressed in T cells (T-bet) [13]. Inappropriate activation of Th1 cells in response to self-antigen or innocuous antigens leads to autoimmune states as well as to hypersensitive states in which T-cell tolerance to environmental antigens fails [14]. 

PPAR*γ* agonists have been shown to decrease IL-2 production in activated T cells and thereby to enhance apoptosis. The modulation of T-cell activity is due to inhibition of IL-2 production in T-cell-receptor-stimulated Th cells and due to suppression of Th2 cell differentiation. The endogenous ligand 15-deoxy-Delta12, 14-prostaglandin J2(15d-PGJ2), and the synthetic ligand ciglitazone inhibit IL-2 secretion by T-cell clones in murine cells [6, 15]. High amounts of IL-2 and IFN-*γ* were detected in the supernatant of antigen stimulated splenocytes from PPAR*γ*+/– mice [16]. Studies demonstrated that lymphocyte-derived IFN-*γ* interferes with PPAR*γ* ligand regulation of MAPK activation in murine macrophages *in vitro *[17]. PPAR*γ* ligands decreased the level of IFN*γ* production in splenocytes and T-cell clones isolated from SJL mice [15]. Treatment with pioglitazone changes the helper T-cell balance from Th1 to Th2 in the myocardium of rats with autoimmune myocarditis by upregulating the mRNA of Th2 cytokine IL-4 and by reducing the mRNA level of Th1 cytokine IFN-*γ* [18]. Pioglitazone also reduced IFN-*γ* production in a model of experimental autoimmune encephalomyelitis (EAE), the inflammatory demyelinating disease model of multiple sclerosis (MS) [19]. *In vivo *treatment with the PPAR-ligand THR0921 resulted in reduced production of TNF-*α*, IL-1*β*, and INF-*γ* by spleen cells cultured for 48 h with either lipopolysaccharide (LPS) or type II collagen (CII) compared with cells from vehicle-treated collagen-induced arthritis (CIA) mice [20]. PPAR*γ* agonists decrease lupus-related nephritis through decreased IFN-*γ* and nitric oxide production in MRL/lpr mice *in vivo *[21]. Treatment of diabetic mice with rosiglitazone resulted in a significant decrease in the pancreatic level of TNF-*α* and IFN-*γ* compared to untreated diabetic mice [22].

In human cells, it has been demonstrated that nuclear factor of activated T cells (NFAT) is negatively regulated by PPAR*γ* activation with troglitazone and 15d-PGJ2 through blockade of NFAT DNA binding and transcriptional activity and subsequent inhibition IL-2 production [23]. IL-2 protein expression was also downregulated by rosiglitazone [24]. The endogenous PPAR*γ* agonist 13-hydroxyoctadecadienoic acid (13-HODE) downregulated IL-2 production by human peripheral blood T lymphocytes by reducing NFAT and NF-*κ*B binding to the IL-2 promoter [25]. In PBMCs from patients with Hashimoto's thyroiditis (HT) and controls, rosiglitazone reduced IFN-*γ* expression by CD4+ and CD8+ T lymphocytes in a dose-dependent manner, but the degree of inhibition was significantly greater in healthy subjects than patients with HT. This *in vitro* resistance to immunomodulation might be due to the enhancement of mitogen-activated protein kinase (MAPK) pathway [26]. The CXC chemokines (CXCL9, CXCL10, and CXCL11), inducible by IFN-*γ*, are proinflammatory molecules with chemoattractant activity for Th1 lymphocytes secreting IFN*γ* [27]. Rosiglitazone has recently been shown to inhibit IFN-*γ* and TNF induction of *α*-chemokine CXCL10 release by cultured thyroid cells and orbital fibroblasts from patients with Graves' ophthalmopathy [28]. Troglitazone has been demonstrated to modulate the level of IFN-*γ* production [29, 30]. The deletion of PPAR*γ* in CD4+ T cells results in enhanced antigen-specific proliferation and overproduction of IFN-*γ* in response to IL-12 highlighting the importance of expression of PPAR*γ* in CD4+ T cells in downregulating excessive Th1 responses [31]. 15d-PGJ2 suppressed T-cell proliferation and IFN-*γ* secretion* in vitro *by both Con A- and myelin basic protein (MBP) Ac1–11 peptide-stimulated lymphocytes. MBP is used to induce EAE in rodents. The ability of T cells to adoptively transfer EAE is suppressed when these cells are cultured with 15d-PGJ2 *in vitro *[32]. 15d-PGJ2 acts cooperatively with 9-*cis *retinoic acid, the ligand for the retinoid X receptor (RXR), in inhibiting microglial cell activation. Microglia participate in pathology associated with multiple sclerosis (MS) [33]. The PPAR*γ* ligands, 15d-PGJ2, troglitazone, and pioglitazone, can inhibit the IFN-*γ*-induced expression of the CXC chemokines inducing protein-10 and monokine induced by IFN-*γ*/IFN-inducible T-cell *α*chemoattractant by endothelial cells [34]. In addition, T-cell-specific PPAR*γ*-deficient mice are suggested to be defective in accumulating T effector cells in secondary lymphoid organs and tissues and therefore in their ability to produce IFN-*γ* gamma and IL-17 in inflammatory sites [26, 35]. 

IL-12 plays a crucial role in the differentiation of T lymphocytes and immunity against pathogens. The development of EAE was also found to be associated with an increase in the expression of IL-12 in the central nervous system (CNS) and lymphoid organs [36, 37]. The PPAR*γ* agonists 15d-PGJ2 and ciglitazone inhibit EAE by blocking IL-12 production in macrophage and microglial cells, IL-12 signaling, and Th1 cell differentiation [38, 39]. The endogenous ligand 9-hydroxyoctadecadienoic acid (9-HODE), a major oxidized lipid component of oxLDL, significantly inhibited IL-12 production in lipopolysaccharide- (LPS-) stimulated mouse macrophages and also suppressed NF-*κ*B-mediated activation in IL-12 p40 promoter [40]. 

The inhibition of IL-12 production by dendritic cells through ligand-activated PPAR*γ*, as well as the inhibition of IFN*γ* production by T cells, indicates that this nuclear hormone receptor might be involved in the differentiation of naive T cells into their effector subsets. These data highlight that PPAR*γ* play important roles in Th1-cell survival, activation, and differentiation. 

## 3. Th2 Cells

Th2 cells classically mediate host defense against extracellular parasites. They are also important in the induction and persistence of asthma and other allergic inflammatory diseases. Th2 cells can produce IL-4, IL-5, IL-9, IL-10, IL-13, and IL-25. IL-4 plays a positive feedback for Th2 cell differentiation through the transcription factor STAT-6 and expression of GATA-3 [41]. Although Th2 cells are not major effectors in the pathogenesis of most autoimmune diseases, in some instances induction of a Th2 response during ongoing autoimmune inflammation can be of therapeutic value, especially considering the potential of Th2 cells to modulate the generation of Th1 cells and their interactions with B cells. Th2 cytokines can stimulate proliferation, activation, and isotype switching of B cells and aid in the production of autoantibodies by providing help to autoreactive B cells [42, 43]. Futhermore, Th2 cytokines, like IL-5, can promote induction of Ag-specific Tregs, contributing to restore autoimmune tolerance [44].

Studies of gene expression have shown that polarized Th2 cells express greater levels of PPAR*γ*2 mRNA than Th1 cells [45]. The exact interaction between PPAR*γ* and IL-4 is not fully understood, and it seems to depend on the context and on the ligand type involved. Treatment with pioglitazone increased expression levels of IL-4 in a model of autoimmune myocarditis, and there was an amelioration of the inflammation [18]. This report agrees with previous description of improvement of acute colitis after thiazolidinic treatment (troglitazone, pioglitazone, and rosiglitazone) by decreasing TNF*α* and IFN*γ* and increasing IL-4, IL-10, and transcription factor GATA-3 expression [46, 47]. Recently, pioglitazone attenuated the neurological signs in a model of experimental autoimmune neuritis in rats by the inhibition of Th1 cytokines production (TNF*α* and IFN*γ*) and increased secretion of IL-4 [48]. In PBMC from Hashimoto's thyroiditis patients, rosiglitazone produced no inhibitory effect on IL-4 expression by CD4+ T lymphocytes [26]. 

On the other hand, significant inhibition of IL-4 production in T cells by natural and synthetic PPAR*γ* agonists (15d-PGJ2 and ciglitazone) was reported [7]. In this study, the inhibitory effect was explained, at least in part, by downregulation of NF-AT (nuclear factor of activated T cells) activation, another proinflammatory signal transduction pathway [7]. This finding was subsequently confirmed by other authors [49, 50]. Furthermore, it was demonstrated that a nonthiazolidinedione PPAR*γ* ligand (KR62980), but not rosiglitazone, decreased IL-4, IL-5, and IL-13 levels and Th2 cell differentiation *in vitro*, by reducing the expression of c-Maf, a Th2-specific transcription factor [51]. These findings suggest that PPAR*γ* activation could have an anti-inflammatory effect on Th2-mediated diseases.

It was also demonstrated that IL-4 and IL-13 could upregulate PPAR*γ* gene expression in CD4+ T cells, peripheral monocytes, peritoneal macrophages, and airway epithelial [52–55]. These authors also showed that IL-4 induces the expression and activity of 12/15-lipoxygenase, enzyme that catalyzes the synthesis of the PPAR*γ* ligands 12-HETE, 15-HETE, and 13-HODE. This finding reinforces the important role of this cytokine in inflammation by coordinately inducing the expression of PPAR*γ* receptor and its ligands and, consequently, proinflammatory gene repression [52, 53]. 

IL-10 is an anti-inflammatory cytokine that downregulates cellular immunity and allergic inflammation, by inhibiting activation and effector function of T cells, monocytes, and macrophages; downregulating IL-4 and IL-5 expression by T-helper type 2 cell lymphocytes and decreasing eosinophil survival and IgE synthesis [56, 57]. In a mouse model of asthma, the administration of rosiglitazone or pioglitazone increased IL-10 levels in lung tissue and decreased IL-4 and IL-5 levels, indicating a protective role for the receptor in inflammatory diseases [50]. The PPAR*γ* agonists effects seem to depend on dose and cell type, since low concentrations of rosiglitazone induced production of IL-10 from mature dendritic cells and activated CD4+ T cells, but these effects were not identified with higher doses or in immature cells. This production was mediated by PPAR*γ* receptors, and it was also described as a functional PPRE in the IL-10 gene promoter [58]. 

Some studies have suggested that PPAR*γ* agonists may also have some proinflammatory activity in which 15d-PGJ2 can inhibit IL-10 action by blocking STAT1 and STAT3 activation. This inhibition was not specific for IL-10, as STAT activation by IFN*γ* or IL-6 is also inhibited by the compound [59]. Thus, some PPAR*γ* ligands can exert their pro- or anti-inflammatory properties through a PPAR*γ*-independent way.

IL-33 can act directly on Th2 cells increasing the secretion of Th2 cytokines such as IL-5 and IL-13 and can also act as a chemoattractant for Th2 cells [60, 61]. It was demonstrated that treatment with PPAR*γ* agonists (15d-PGJ2 and rosiglitazone) could also reduce the production of IL-33, and they have been implicated in the pathogenesis of some inflammatory diseases mediated by eosinophils, like asthma [62]. 

In addition to downregulating Th1 proinflammatory cytokines, PPAR*γ* ligands can presents anti-inflammatory effects by promoting the production of anti-inflammatory Th2 cytokines. Thus, PPAR*γ* was suggested to modulate the orientation of immune responses in favor of Th2 responses, but the studies are not uniform.

## 4. Th17 Pathway

Recently, a new subset of Th cells has been identified named TH17 cells and characterized by the production of IL-17A, IL-17F, IL-21, IL-22, and IL-23R. Th17 cell differentiation is enhanced by the coordinated functions of distinct cytokines including TGF*β*, IL-6, IL-21, and IL-23, whereas IL-2, IL-4, IFN*γ*, and IL-27 inhibit its differentiation. The IL-17A and IL-17F induce proinflammatory cytokines like IL-6, IL-1, TNF, and proinflammatory chemokines like CXCL1, GCP-2, and IL-8 and thus promote tissue inflammation and recruitment of neutrophils to the site of inflammation [63]. This cell population has been implicated in the development of autoimmune diseases, such as multiple sclerosis, rheumatoid arthritis, and inflammatory bowel disease and has been studied in mouse models of autoimmunity, such as experimental autoimmune encephalomyelitis, inflammatory bowel disease, and collagen-induced arthritis [64–67]. In several autoimmune diseases, Th17 cells are recruited to inflamed tissues and promote inflammation by enhancing cytokine production, which can in turn activate B-cell antibody production, activate dendritic cells, and stimulate resident cells in the target tissues [57]. 

Pharmacological PPAR*γ* activation selectively impairs differentiation into Th17 cells. Under physiological conditions, the corepressor SMRT (silencing mediator of retinoid and thyroid hormone receptors) is bound to the ROR*γ*t promoter and inhibits its transcription. PPAR*γ* activation is thought to prevent removal of this corepressor complex, thus suppressing ROR*γ*t expression and ROR*γ*t-induced Th17 cell differentiation [67, 68]. In PPAR*γ* knockout mice (PPAR*γ* −/−), Th17 differentiation was strongly increased. In a model of EAE, characterized by increased infiltration of Th17 cells into the central nervous system, pioglitazone treatment alleviated the disease severity of EAE, and PPAR*γ* −/− mice were reported to exhibit enhanced disease severity. In CD4+ T cells isolated from the central nervous system (CNS) of these EAE mice, endogenous (13-HODE) and synthetic (pioglitazone) PPAR*γ* agonists suppressed Th17 differentiation, but not Th1, Th2, or Treg differentiation. A decreased expression of Th17 cytokines IL-17A, IL-17F, IL-21, IL-22, and IL-23R and a selective inhibition of TGF*β*/IL-6-mediated expression of ROR*γ*t were also demonstrated [69]. 

IL-23 belongs to IL-12 cytokine family and represents an important cytokine implicated as being responsible for Th17 phenotype maintaining and survival [70, 71]. PPAR*γ* agonists (15d-PGJ2 and rosiglitazone) inhibited the induction of IL-23 protein by LPS-stimulated CNS cells [72]. In models of allergic asthma, treatment with PPAR*γ* agonists (15d-PGJ2 and rosiglitazone and pioglitazone) promoted the reduction of IL-17 and IL-23 [62, 73]. These studies demonstrate that PPAR*γ* activation can regulate the differentiation and function of Th17 cells, by suppressing Th17 cell development and decreasing Th17 cytokines.

## 5. T Regulatory (Treg) Cells

Treg cells suppress autoimmune responses and also other aberrant or excessive immune responses to nonself-antigens. Depletion of CD25+CD4+ Treg cells, which constitute 5%–10% of CD4+ T cells, produces autoimmune diseases such as inflammatory bowel disease in normal mice [74]. Expression of PPAR*γ* by macrophages and epithelial cells is required for protection against dextran sodium sulfate colitis [75, 76]. These Treg cells express FoxP3, a transcription factor essential for their development and function [77]. PPAR*γ*-expressing Treg effectively reduce IFN-*γ*-producing CD4+ T cells. Therefore, the loss of PPAR*γ* in Treg impairs their ability to control effector CD4+ T-cell responses preventing protection against colitis in a mouse model of intestinal inflammation suggesting that expression of PPAR*γ* by Treg is required for optimal anti-inflammatory efficacy [31]. 

PPAR*γ* deficiency leads to decreased numbers of CD4+Foxp3+ T cells and increased CD4+IFN-*γ*+ cells, suggesting that PPAR*γ* plays a role in Treg survival and regulation of effector T-cell functions. Similarly, T-cell-specific PPAR*γ*-deficient mice showed reduced Treg recruitment to mesenteric lymph nodes and increased expression of apoptosis-related genes [78]. In addition, ciglitazone or PGE2 treatment of naïve CD4+ T cells enhanced induction of Foxp3+ inducible regulatory T cells, suggesting that PPAR*γ* may contribute to the quality and quantity of Treg functions *in vivo*. PPAR*γ* regulates induction of Tregs through retinoic acid-mediated dendritic cells (DCs) [79, 80].

Foxp3+ Treg cells are abundant in visceral adipose tissue and have a different T-cell receptor repertoire compared with Treg cells in other tissues [81]. These cells specifically express the PPAR*γ* and its stimulation by pioglitazone and increase Treg cell numbers in the visceral adipose tissue [82]. These findings suggest that PPAR*γ*-expressing Treg cells in adipose tissue might control inflammation in obesity, providing a new link between immunoregulation and metabolic disease [83]. 

## 6. B Cells

The exact role of B cells in the pathogenesis of autoimmune diseases is still matter of research. One previous hypothesis proposed that autoimmune disease develops as a result of persistence of self-reactive clones of lymphocytes that should have been deleted via normal immune tolerance, although some had suggested that this could be an epiphenomena. The success of B-cell depletion therapy in autoimmune diseases, particularly in rheumatoid arthritis (RA) [84], systemic lupus erythematosus (SLE) [85], antineutrophil cytoplasmic antibody- (ANCA-) related vasculitides [86], and multiple sclerosis [87], reinforces the importance of B cells in the pathogenesis [88]. 

Papers discussing the role of PPAR*γ* agonists in B cells focus on the induced apoptosis by natural and synthetic agonists. The PPAR*γ* mRNA and protein expression in mouse B cells is described, and it has been suggested that PPAR*γ* agonists (15d PGJ2) could be involved in the induction of apoptosis in B lymphocytes [89]. The same group demonstrated the nuclear and cytoplasmic expression of PPAR*γ* in normal human B lymphocytes. An antiproliferative effect of natural (15d PGJ2) and synthetic PPAR*γ* ligands (ciglitazone) on human B cells through inducing apoptosis is also shown [90].

On the other hand, PPAR*γ* expression was increased in human activated B cells as compared to nonactivated cells. Using low doses of PPAR*γ* ligands (15-d-PGJ2 and rosiglitazone), they found an increase of B-cell proliferation and IgM and IgG antibody production. These effects were related to activation of the cells: activated B cells, which had higher PPAR*γ* levels, can respond to PPAR*γ* ligands, while nonactivated B cells, with low PPAR*γ* expression, were not able to activate PPAR*γ* upon low-dose PPAR*γ* ligand exposure. There was also a raise of stimulated memory B cells differentiation to plasma cells [12]. In an animal model of asthma, 15d-PGJ2 inhibited LPS-induced B cell proliferation [62], and, in peripheral blood mononuclear cells of atopic dermatitis patients, there was significant inhibition of IgE synthesis [49]. 

The role of PPAR*γ* in B cells in autoimmune diseases is less well documented. In PPAR*γ* heterozygote knockout mice (PPAR*γ*+/–), in which PPAR*γ* expression is reduced by 50%, B-cell proliferative response was enhanced, but not T cells. Furthermore, PPAR*γ*+/− mice developed more severe antigen-induced arthritis, that was suggested to be due to B-cell hyperreactivity. However, the production of T-cell-derived cytokines was also enhanced, since higher amounts of both IL-2 and IFN-*γ* were detected in the supernatant of antigen stimulated splenocytes from PPAR*γ*+/– mice, and it was suggested that the alteration in T-cell function caused by reduced PPAR*γ* expression could be responsible for the results [16].

The interaction between T and B cells and the autoantibodies production are key elements in the SLE pathogenesis. Recently, some studies have suggested the PPAR*γ* participation in this complex disease. An increased PPAR*γ* expression in patients with active SLE was described [91]. PPAR*γ* agonist rosiglitazone was shown to reduce autoantibody production and ameliorate renal disease in a murine SLE model [92]. Ciglitazone inhibited IgE production in nonallergic and atopic dermatitis models *in vitro* and *in vivo* [49]. Indeed, these effects are not proven directly mediated by activated B lymphocytes, but rather indirectly mediated via regulatory signal pathways of other cells types. Reduction in PPAR*γ* expression increases T-cell proliferation and skews toward Th1 immune response, which includes increased IFN*γ* and IL-12 production [16, 38]. These cytokines can directly influence B-cell function, including plasma cell formation, proliferation, and antibodies production [93–95]. 

## 7. Perspectives

**Figure 1:**
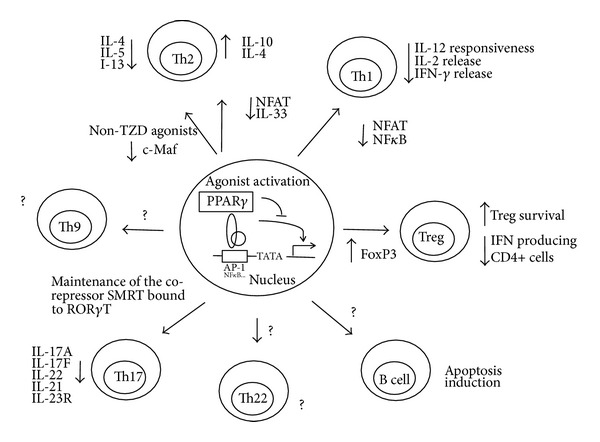
Effects of PPAR*γ* agonists on cytokine expression and on lymphocytes involved in adaptive immunity. ? Represents mechanisms that are not well elucidated.

In conclusion, PPAR*γ* agonists are important modulators of the inflammatory process and lymphocyte homeostasis. Currently, there is evidence to support that PPAR*γ* is involved in Th lymphocyte differentiation, B lymphocyte effector functions, and cytokine expression.  Figure 1 summarizes the effects of PPAR*γ* agonists on cytokine expression and on T regulatory cells and B cells. PPAR*γ* is expressed by the main cell types of adaptive responses. Natural and synthetic PPAR*γ* ligands proved to be capable of inhibiting major signaling pathways of adaptive immunity, reducing or augmenting the expression of cytokines. In fact, PPAR*γ* ligands were shown to inhibit the production of several proinflammatory cytokines. Thus, further studies are necessary to clarify the use of PPAR*γ* antagonists in diseases driven by the Th imbalance such as autoimmune diseases. The actions of these compounds at the cellular levels and their proven immunomodulatory effects make it worth considering their use in clinical trials exploring the possibilities that these drugs might help in the treatment of immune diseases.
